# Careful Consideration of Study Limitations When Interpreting the Association Between Induced Abortion and Breast Cancer Risk

**DOI:** 10.2188/jea.JE20230362

**Published:** 2024-10-05

**Authors:** Tiana Fontanilla

**Affiliations:** Office of Public Health Studies, University of Hawaiʻi at Mānoa, Honolulu, Hawaii

To the editor:

In 2018, Yuan et al reported findings from a case-control study that investigated the impact of induced abortion and birth control methods on breast cancer risk, respectively. The authors stated that the results included “significant effects of exposure of medical abortion and surgical abortion breast cancer risk among both pre-menopausal and post-menopausal women”.^1^ However, the authors should have been more cautious in their interpretation of the measures of association for abortion and breast cancer risk given key limitations of their study, some of which were not discussed in the article.

The first limitation not adequately discussed was the lack of specificity or detail of the exposure measures. For example, type of intrauterine device (hormonal vs non-hormonal), number of alcoholic drinks consumed within “one time”, dose and frequency of oral contraceptives taken, number of cigarettes or other substance smoked, degree of second-hand tobacco smoke exposure, and time of induced abortion(s) were not collected from participants. These measures could have been better defined and more specific to minimize the risk of misclassification and draw more meaningful conclusions. Other commonly known risk factors could have been queried, such as family history of breast cancer or prior exposure to carcinogenic substances.

The second limitation that should have been highlighted was the self-reporting of measures by participants to interviewer staff, which means there was potential social desirability bias and, more importantly, recall bias, a common disadvantage of case-control studies. This limitation cannot be taken lightly. Per the American College of Obstetricians and Gynecologists, while prospective studies show that there is no association between induced abortion and breast cancers, early case-control studies did report an association but “had significant methodological problems, most notably reliance on retrospective reporting of abortion history”.^2^ Of note, a meta-analysis of 83,000 women worldwide that examined the relationship between induced abortion and breast cancer found significant differences in the overall relative risks for studies involving prospective and retrospective collection of abortion history, respectively,^3^ suggesting that reporting bias likely impacted studies where this information was collected retrospectively.

Furthermore, the regression model examining the association between induced abortion and breast cancer produced extremely wide confidence intervals, particularly among the post-menopausal subgroup (crude odds ratio [OR] 9.00; 95% confidence interval [CI], 1.12–72.44 and adjusted OR 17.14; 95% CI, 1.96–149.89 for the odds of breast cancer among participants exposed to both medical and surgical abortions relative to those exposed to neither type of induced abortion), suggesting considerable uncertainty of the true odds ratios.

Given the potential healthcare implications of this research and the known challenges of retrospective studies, the authors should have thoroughly acknowledged all study limitations and more cautiously interpreted their measures of association between induced abortion and breast cancer risk.

Conflicts of interest: None declared.
